# Community-Based Public Health Vaccination Campaign (VaccinateLA) in Los Angeles’ Black and Latino Communities: Protocol for a Participatory Study

**DOI:** 10.2196/40161

**Published:** 2023-02-20

**Authors:** Michele D Kipke, Nicki Karimipour, Nicole Wolfe, Allison Orechwa, Laura Stoddard, Mayra Rubio-Diaz, Gemma North, Ghazal Dezfuli, Sheila Murphy, Ashley Phelps, Jeremy Kagan, Kayla De La Haye, Christina Perry, Lourdes Baezconde-Garbanati

**Affiliations:** 1 Southern California Clinical and Translational Science Institute University of Southern California Los Angeles, CA United States; 2 Department of Pediatrics Keck School of Medicine of University of Southern California Children's Hospital Los Angeles Los Angeles, CA United States; 3 Office of the Senior Vice President of Health Affairs University of Southern California Los Angeles, CA United States; 4 Annenberg School for Communication and Journalism University of Southern California Los Angeles, CA United States; 5 Department of Population and Public Health Science Keck School of Medicine of University of Southern California Los Angeles, CA United States

**Keywords:** community engagement, COVID-19, vaccine, vaccination, public health, population health, health equity, social media, vaccine hesitancy

## Abstract

**Background:**

The COVID-19 pandemic has significantly affected Los Angeles County and disproportionately impacted Black and Latino populations who experienced disparities in rates of infection, hospitalizations, morbidity, and mortality. The University of Southern California (USC), USC Keck School of Medicine, Southern California Clinical and Translational Science Institute, USC Mann School of Pharmacy and Pharmaceutical Sciences, Annenberg School for Journalism and Communication, and Children’s Hospital Los Angeles will launch a collaborative public health campaign called VaccinateLA.

**Objective:**

VaccinateLA will implement a community-based, community-partnered public health campaign that (1) delivers culturally tailored information about COVID-19 and available vaccines; and (2) addresses misinformation and disinformation, which serves as a barrier to vaccine uptake. The campaign will be targeted to communities in Los Angeles with the highest rates of COVID-19 infection and the lowest vaccination rates. Using these criteria, the campaign will be targeted to neighborhoods located in 34 zip codes in the Eastside and South Los Angeles. The primary aim of VaccinateLA will be to design and deliver an evidence-based multimedia public health campaign tailored for Black and Latino populations. A secondary aim will be to train and deploy community vaccine navigators to deliver COVID-19 education, help individuals overcome barriers to getting vaccinated (eg, transportation and challenges registering), and assist with delivering vaccinations in our targeted communities.

**Methods:**

We will use a community-based, participatory research approach to shape VaccinateLA’s public health campaign to address community members’ attitudes and concerns in developing campaign content. We will conduct focus groups, establish a community advisory board, and engage local leaders and stakeholders to develop and implement a broad array of educational, multimedia, and field-based activities.

**Results:**

As of February 2023, target communities have been identified. The activities will be initiated and evaluated over the course of this year-long initiative, and dissemination will occur following the completion of the project.

**Conclusions:**

Engaging the community is vital to developing culturally tailored public health messages that will resonate with intended audiences. VaccinateLA will serve as a model for how an academic institution can quickly mobilize to address a pressing public health crisis, particularly in underrepresented and underresourced communities. Our work has important implications for future public health campaigns. By leveraging community partnerships and deploying community health workers or *promotores* into the community, we hope to demonstrate that urban universities can successfully partner with local communities to develop and deliver a range of culturally tailored educational, multimedia, and field-based activities, which in turn may change the course of an urgent public health crisis, such as the COVID-19 pandemic.

**International Registered Report Identifier (IRRID):**

PRR1-10.2196/40161

## Introduction

The COVID-19 pandemic significantly affected Los Angeles County [1] and disproportionately impacted Black and Latino populations in Los Angeles who experienced disparities in rates of infection, hospitalizations, morbidity, and mortality. [2,3].

In response, the University of Southern California (USC), USC Keck School of Medicine, Southern California Clinical and Translational Science Institute, USC Mann School of Pharmacy and Pharmaceutical Sciences, and Annenberg School for Journalism and Communication, and Children’s Hospital Los Angeles will launch a collaborative year-long public health campaign called VaccinateLA. Given the size and diversity of Los Angeles County (ie, more populous than 42 other US states), we will focus this campaign on communities with the highest rates of infection and the lowest rates of vaccination. Using these criteria, the campaign will be delivered in neighborhoods located in 34 zip codes located in the Eastside and South Los Angeles. The overarching aim of VaccinateLA will be to design and deliver an evidence-based multimedia public health campaign tailored for Black and Latino populations. A secondary aim will be to train and deploy community vaccine navigators (CVNs) to deliver COVID-19 education, help individuals overcome barriers (eg, transportation and challenges registering) to becoming vaccinated, and assist with delivering vaccinations in our targeted communities. In this paper, we describe VaccinateLA, including our planned approach to partnering with the community to develop and implement a broad array of evidence-based educational, multimedia, and field-based activities.

## Methods

### Approach

The overarching goal of VaccinateLA is to implement a community-based, community-partnered public health campaign intended to deliver culturally tailored information about COVID-19 and available vaccines, as well as to address misinformation and disinformation, which can serve as barriers to vaccine uptake. The difference between misinformation and disinformation is intent [4]. Misinformation is erroneous information reported as fact, whereas disinformation is a form of misinformation that is intended to deceive or mislead [4]. The aims of VaccinateLA will be accomplished through a range of public health campaign strategies.

### Conceptual Model

Through VaccinateLA, we seek to strengthen existing partnerships and forge new relationships in our targeted communities, as well as to engage the public health community and other key stakeholders throughout the greater Los Angeles County area (eg, with the Los Angeles County Departments of Public Health, Health Services, and Mental Health).

**Figure 1:**
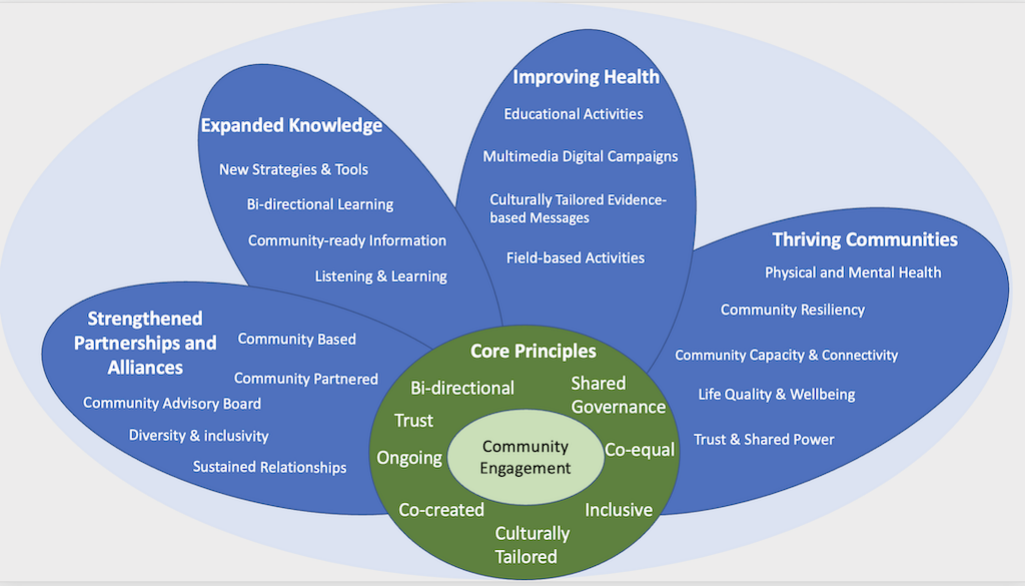
Conceptual model, titled VaccinateLA Public Health Campaign: Building Health Equity Through Community Engagement.

We plan to use a community-based, participatory research approach to shape VaccinateLA’s public health campaign. Our work will be guided by a conceptual model that was adapted from a framework developed by the National Academy of Medicine’s Leadership Consortium: Collaboration for a Value & Science-Driven Health System [5]. Our conceptual model, titled *VaccinateLA Public Health Campaign: Building Health Equity Through Community Engagement*, takes a holistic approach to engaging multiple communities and key stakeholders to promote equity (Figure 1).

Our core principles of community engagement include work that is bidirectional, inclusive, culturally tailored, and co-equal and has shared governance to build trusting relationships. When developing a public health campaign, the work should be cocreated and ongoing. Our goals are to *strengthen community partnerships and alliances* and *expand our knowledge* through bidirectional learning (eg, listening to community members and learning from them about their experiences and needs will guide our work), with the goal of being able to develop effective, resonant, and community-ready messages, strategies, and tools. We will be able to succeed through the sustained relationships that are developed through this process, which includes a diverse and inclusive community advisory board (CAB). VaccinateLA intends to develop culturally tailored evidence-based messages and a multimedia digital educational campaign and launch education and field-based activities to *improve the health of our targeted communities*. Trust and shared power will help guide this community-based, community-partnered initiative, resulting in our ability to promote *thriving communities*. By engaging multiple communities and key stakeholders, we can increase community connectivity and the capacity to develop and sustain beneficial public health programs that address physical and mental health, ideally improve quality of life and overall well-being, and ultimately support and enable community resiliency

### Description of Our Targeted Communities and Populations

#### Community Overview

**Figure 2:**
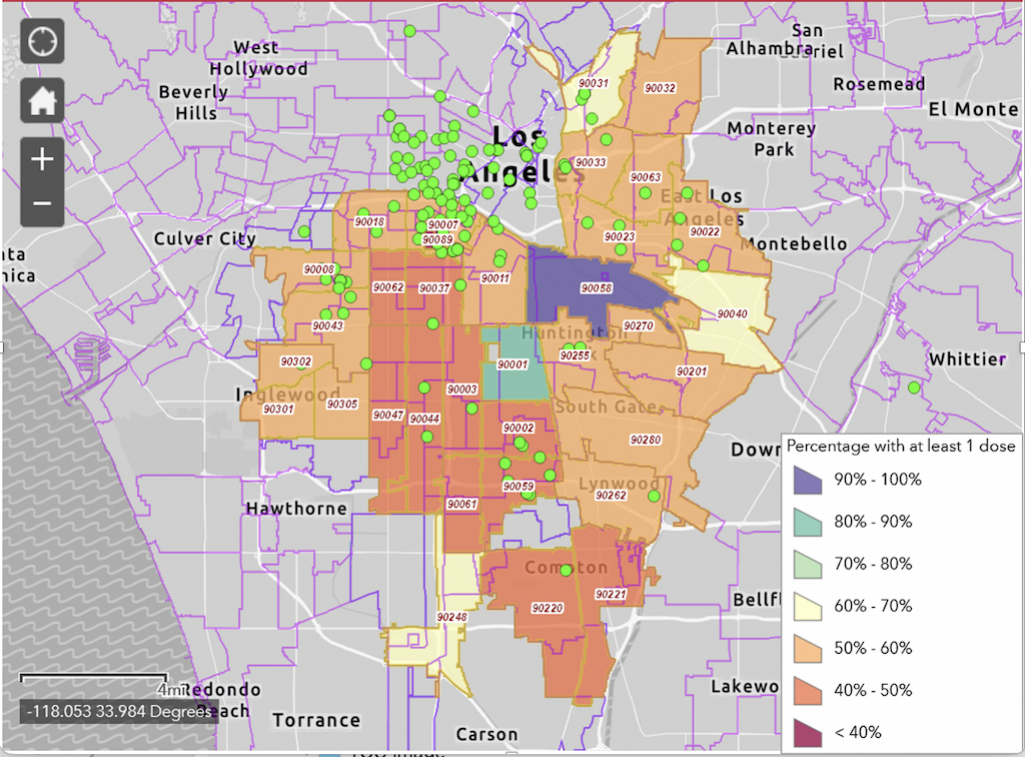
VaccinateLA map of targeted zip codes and vaccination rates. The green dots represent our 160 community partners.

Los Angeles County is a large and diverse county that is more populous than 42 states in the United States. In Los Angeles County, 185 languages are spoken and 42% of residents are Spanish speakers [6]. Given the sheer size of Los Angeles County, we plan to target the campaign to communities with the highest rates of infection and the lowest vaccination rates. Using these criteria, we identified 34 zip codes located in the Eastside and South Los Angeles. A map of neighborhoods in these zip code areas (based on vaccination rates from May 2021) is shown in Figure 2. The goal will be to work closely with existing partners and forge new partnerships in these same communities. The campaign will target to five distinct segments of the population: (1) Latino and Black residents of South Los Angeles; (2) Latino and Black residents in the Eastside of Los Angeles; (3) members of the lesbian, gay, bisexual, transgender, and queer (LGBTQ+) community; (4) parents of school-age children, both vaccinated and unvaccinated; and (5) community health workers in Spanish-speaking communities, called *promotores de salud*.

#### South Los Angeles Community

This area has historically been predominantly Black; however, approximately 28% of current South Los Angeles residents are Black. Low-wage, temporary jobs are common in this area, as is poverty and unemployment [7].

#### East Los Angeles Community

East Los Angeles is historically a predominantly Latino area, and approximately 96% of current residents in this area identify as Hispanic or Latino. Spanish is the first language for most residents aged 5 years and older [8]. The median household income is just over US $46,000, and approximately 20% of the population live at the poverty line [8].

#### Parents

The pandemic’s effect on parents included hardships such as facilitating online learning, new safety measures at school, and an overwhelming amount of information about vaccine and booster eligibility. Balancing multiple priorities at work and at home, caring for sick family members, and experiencing loss and unemployment also take a toll.

#### LGBTQ+ Community

The LGBTQ+ community is also facing challenges and barriers to getting vaccinated. In California, 5.3% of the population identify as gender and/or sexual minority individuals [9]. Reliable statistics for county-level data could not be identified. These individuals regularly experience challenges in accessing medical care, are often stigmatized and discriminated against, and experience high rates of violence [10].

#### Community Health Workers

Our final group of interest is *promotores de salud* [11]. We will partner with an organization, called Vision y Compromiso, which provides comprehensive and ongoing leadership development, capacity building, advocacy training, and support to over 4000 *promotores* throughout the State of California and in a region within Mexico [12]. We will conduct a focus group with *promotores* to understand the challenges that local communities experience during the pandemic.

### VaccinateLA Public Health Campaign Strategies

As a starting point, we will first establish a CAB and then initiate a *listening tour* using a semistructured interview guide to listen to and learn from the community. Then, in partnership with the community, we will develop and deliver a combination of *educational* (town halls and workshops), *multimedia* (radio, local newspapers, social media campaigns, and project website), and *field-based activities* (pop-up vaccination clinics, deployment of a cadre of embedded community workers, and restorative circles). The following provides a description of each of these activities, the sequencing of which can be found in Table 1.

**Table 1:** Project timeline.

Activity	Month											
	1	2	3	4	5	6	7	8	9	10	11	12
Develop VaccinateLA website	✓^a^											
Community advisory board	✓	✓	✓	✓	✓	✓	✓	✓	✓	✓	✓	✓
Conduct focus groups	✓	✓	✓	✓						✓	✓	
Town halls	✓	✓			✓			✓	✓	✓		
Educational workshops		✓	✓	✓	✓	✓	✓	✓	✓	✓	✓	✓
Print and digital fotonovela				✓	✓							
Culturally tailored short films		✓	✓	✓	✓							
#ShareYourWhy testimonials		✓	✓			✓	✓			✓	✓	✓
Public service announcements									✓	✓	✓	✓
Social media campaign		✓	✓	✓	✓	✓	✓	✓	✓	✓	✓	✓
Pop-up vaccination clinics	✓	✓	✓	✓	✓	✓						
Community vaccine navigators	✓	✓	✓									
Restorative circles			✓	✓	✓							

^a^✓: denotes the timepoints at which these activities will be completed.

### Establishing a CAB

The CAB will consist of 10-15 members, including trusted and respected community leaders, social service providers, and residents from our targeted communities. The CAB will include both men and women and a diverse racial/ethnic representation; the CAB will be chaired by a member of the board. The CAB will meet virtually on a monthly basis or more, based on what is needed. CAB members will receive an electronic gift card in the amount of US $25 to compensate them for their time and effort. The aim of the CAB is to provide valuable insight and important feedback to inform our outreach activities, multimedia educational materials, and dissemination strategies. CAB members will also help introduce us to new community partners and provide guidance on how to liaise with the community. Representatives from the Los Angeles County Department of Public Health and the Los Angeles Unified School District will also be invited to serve on the CAB.

### Focus Groups to Listen to and Learn From the Community

We will begin by conducting a series of focus groups with various segments of the community to understand sources of concern, vaccine hesitancy, and barriers to becoming vaccinated. We will then conduct additional focus groups to inform our educational campaign. These focus groups will be conducted with various segments of the population, including community leaders and key stakeholders, young adults, older adults, members of the LGBTQ+ community, parents of vaccinated and unvaccinated children, community health workers in the Latino community (ie, *promotores de salud*), and community residents from 2 large public housing developments.

We plan to conduct a total of 40 to 45 focus groups, each with 7 to 8 participants. These focus groups will be conducted in both English and Spanish depending on the primary language of the participants. The groups will be conducted virtually using a semistructured interview guide with questions that focus on barriers, misinformation, and experiences at vaccination sites. These sessions will last 60-120 minutes. Following each session, discussions and comments will be transcribed, deidentified, and coded by researchers trained in qualitative research. Findings will then be summarized to inform real-time, culturally tailored educational campaign messages. Focus group participants will receive US $50 to compensate them for their time and effort.

### Educational Activities

#### Town Halls

A series of virtual townhall events for community members will be delivered. The format will be to have 1 or 2 guest speakers offer short presentations (approximately 10 minutes), followed by up to 60 minutes for questions and answers. Our guest speakers will include trusted community leaders, local physicians, pediatricians, and other health care providers. Guest speakers will provide up-to-date information about the pandemic, infection rates in local communities, and the safety and efficacy of available vaccine/boosters. Information about available resources will also be presented (eg, how and where to access vaccinations/boosters). Town hall events will also be offered to specific segments of the population, including parents of children eligible for the vaccine and booster, Spanish-speaking parents, members of the LGBTQ+ community, and congregations within the faith community. We will advertise and promote these events through email listserves, targeted outreach to community partners, flyers, and social media. The flyer will include information about how to RSVP online, as well as how to submit questions in advance of the event. After each townhall event, we will develop frequently-asked-question sheets based on the questions asked by the community.

#### Workshops

Educational workshops will be offered virtually to smaller groups, allowing for more focused discussions as well as more time allotted to address attendees’ questions/concerns. These workshops will be delivered by *promotores* on our staff. Workshops will be offered multiple times per week and regularly updated to reflect the most recent data and information regarding vaccine and COVID-19 regulations and requirements. Due to the skills and background of our *promotores*, workshops can be developed quickly to meet the evolving educational needs of the community. Attendees will be asked to provide feedback after each workshop to help inform future workshop topics.

### Multimedia Digital Educational Campaign

#### Overview

We will work with local artists, content creators, and film makers to develop and produce a variety of still images and messages for social media, public service announcements (PSAs), brief #ShareYourWhy testimonial videos, short films, and a fotonovela. In addition to partnering with the local community, we will also be partnering with Wondros, a creative design and production agency, to help us craft engaging messages to address common fears, misinformation, and questions about the safety and efficacy of the vaccines. Once developed, we will solicit input from community leaders and CAB members before the products are disseminated. With each product, individuals will be directed to the VaccinateLA website for more information [13]. Most importantly, the website will also include links to additional information and resources, such as a list of local vaccination sites, a link to make a vaccination appointment, and a way to request assistance from a vaccine navigator. The following provides more information about the planned multimedia campaign.

#### Print and Digital Fotonovela

We will work with the USC Mann School of Pharmacy and Pharmaceutical Sciences to develop a bilingual fotonovela entitled *Infectious Rumors*. We will partner with La Opinión, a popular local newspaper, to print and distribute 40,000 copies to their subscribers as an insert in their paper; an additional 10,000 copies will be distributed to community partners, pediatric offices, and schools and at vaccine pop-up clinics. Fotonovelas are print versions of telenovelas, which use storytelling as an educational tool and are popular among Latino populations [14].

#### Culturally Tailored Short Films

We will partner with the USC’s Annenberg School for Journalism and Communication and USC School of Cinematic Arts to produce two 8-minute films. These will be coproduced and directed by local Black and Latino filmmakers, who will assist with storyboarding, planning, filming, and editing. One film, called *Happy Birthday Granny*, will feature a birthday celebration of a Black family. A second film, called *Of Reasons and Rumors*, will feature 2 young Latinas discussing rumors and misinformation associated with the vaccine (in both English and Spanish). Both films will use the art of storytelling as an approach to deliver evidence-based information about COVID-19, routes of transmission, and the safety and efficacy of the vaccine, as well as to address common misinformation and disinformation about COVID-19 and the vaccines. The videos will be posted on the VaccinateLA’s website, on project-dedicated YouTube and Vimeo accounts, on social media accounts created for VaccinateLA, and with organizations such as the American Association of Public Health and the National Alliance for Hispanic Health with its 15 million Latino members nationwide.

#### #ShareYourWhy Testimonials

Local leaders, community members, physicians, nurses, and pediatricians will be asked to share their experiences and reasons for why they chose to be vaccinated; these testimonials will be recorded and edited into short 1-minute videos, called #ShareYourWhy. The #ShareYourWhy videos will be produced by a local video production and editing company, called Everyone Can Eat.

#### PSAs

We will partner with USC’s Hollywood, Health & Society to produce multiple PSAs. USC’s Hollywood, Health & Society will also partner with LifeNoggin, to develop an animated video to be titled, *What if everyone got the COVID vaccine?* LifeNoggin is a YouTube channel that uses animation to deliver scientific content to its more than 3.26 million subscribers.

#### Social Media Campaign

The VaccinateLA team, with assistance from Wondros and Everyone Can Eat, will launch a full-scale social media campaign on Facebook, Instagram, Twitter, and YouTube. We will create original content in both English and Spanish using still images, reels, and short videos with captions and video end cards directing individuals to our website to learn more or schedule an appointment. Data analytics will be reviewed to ensure we successfully reach our targeted audiences

### Field-Based Activities

#### Pop-up Vaccination Clinics

We will partner with the USC Mann School of Pharmacy and Pharmaceutical Sciences, to offer pop-up vaccination clinics in locations chosen by the community (eg, church parking lots, health fairs, local YMCAs, places of employment, and other local venues). At pop-up clinics, we also will host food banks, back-to-school backpack distributions, and tables with information and access to resources such as housing assistance and employment. We will coordinate with social services, such as Women, Infants, and Children and rent relief resources, to distribute essential products such as diapers, personal protective equipment, and personal hygiene and household products such as detergent and shampoo. In addition, we will provide preventive health screenings such as blood pressure screenings and wellness counseling. Members of our team and CVNs will staff these events. Toolkits with advice on how to replicate such pop-up vaccine clinics will be developed and available on the VaccinateLA website.

#### CVNs in Targeted Communities

We will train and deploy community-embedded health workers and *promotores* to be CVNs in our targeted communities. This virtual training will involve 6 to 8 hours of teaching and instructor-led educational content over multiple days, culminating in a 4.5-hour experiential field training. CVNs will learn about COVID-19 transmission, risk mitigation, how to identify and refer people to credible sources, vaccine efficacy, and how to schedule vaccine appointments on someone’s behalf. At the end of their training, each CVN will receive a toolkit to reinforce the training. In South Los Angeles, we will partner with the Southern California National Council of Negro Women, Inc. to deploy CVNs. We will be partnering with Vision y Compromiso to train and deploy CVNs in our targeted Latino communities.

#### Restorative Circles

We intend to conduct in-person restorative circles facilitated by community partners with Black men aged 18 to 35 years and individuals who identify as LGBTQ+. A restorative circle is an intentional gathering of people who have a shared, often traumatic or painful, experiences. These circles serve as a safe space for sharing struggles and expressing grief and trauma, ideally providing a path to healing and connection with others who may have had similar experiences. They are designed to address the longstanding historical trauma and pain around topics such as medical and institutional racism and police brutality against people of color and gender and sexual minority groups, which have worsened and been made more visible during the pandemic. Meals will be provided, and each participant will be offered a parting gift designed by the facilitator, such as journals, pens, and additional information about self-care, mental health, and domestic services in the Los Angeles area.

### Ethics Approval

Data collection protocols for the proposed qualitative focus group interviews have received approval from Children’s Hospital Los Angeles’ Institutional Review Board (CHLA-21-00152). All participants will be identified through community outreach; screened for eligibility; and if eligible, invited to participate. All participants will provide written informed consent using DocuSign during a virtual consenting visit with project staff. Participants will be consented in both English and Spanish and will be provided a hardcopy of their signed consent form.

## Results

As of February 2023,target communities have been identified. The activities will be initiated and evaluated over the course of this year-long initiative, and dissemination will occur following the completion of the project. The activities will be sequenced according to the project timeline in Table 1.

## Discussion

Universities are in a unique position to leverage their immense resources and partner with local communities to respond to emerging community and public health crises [15]. This is especially true in underresourced urban communities [15]. To build trust, it is critical to develop meaningful partnerships with local leaders and other key stakeholders. It is through these partnerships and authentic community engagement that public health messages can be developed and delivered in a culturally tailored and culturally responsive way [16].

Our work and the VaccinateLA campaign will build upon a growing literature that describes best practices to forging academic-community partnerships. This literature demonstrates that local communities welcome the opportunity to partner with academic, health, and public health institutions if the goal is to improve community health [16,17]. Moreover, our work will be grounded in a framework recently developed by the National Academy of Medicine [5].

VaccinateLA will be a true academic-community–partnered initiative designed to respond to a public health crisis. A crucial first step will be to listen to and learn from the community. Findings from a variety of data-gathering activities (ie, focus groups, feedback during workshops, and the CAB) will in turn be analyzed and used to help determine *who the right messengers are*, *what are the right messages*, and *how these messages should be delivered* to reach individuals living and working in our targeted, high-impact communities. We will evaluate this effort to determine if VaccinateLA’s educational and multimedia campaigns were successful at reaching our intended audience, encouraging individuals to become vaccinated, and if CVNs and field-based activities helped to reduce practical barriers to becoming vaccinated. Ultimately, we hope to demonstrate that VaccinateLA did successfully increase rates of vaccinations in our targeted communities.

It is our hope that the proposed VaccinateLA initiative might also have important implications for other public health campaigns. Indeed, we believe that VaccinateLA will serve as a model for how academic institutions can quickly mobilize and respond to a pressing public health crisis, particularly in underrepresented and underresourced communities. Additionally, we hope to demonstrate that such an academic-community partnership can successfully engage with and be responsive to the local needs in underresourced communities. Furthermore, it is our hope that VaccinateLA will serve as a model for how to develop and deliver culturally tailored evidence-based messages that might also be generalizable and potentially used to address other acute public health crises and chronic health conditions (eg, cancer screening, diabetes prevention and care, and asthma prevention).

## Abbreviations

CAB: community advisory board
CVN: community vaccine navigator
LGBTQ+: lesbian, gay, bisexual, transgender, and queer
PSA: public service announcement
USC: University of Southern California

## References

[1] (2022). LA County daily COVID-19 data. County of Los Angeles Public Health.

[2] (2022). Risk for COVID-19 infection, hospitalization, and death By race/ethnicity. Centers for Disease Control and Prevention.

[3] (2021). Tracking COVID-19. California Department of Public Health.

[4] Carsten Stahl B (2006). On the difference or equality of information, misinformation, and disinformation: a critical research perspective. Informing Sci J.

[5] (2022). Assessing meaningful community engagement: a conceptual model to advance health equity through transformed systems for health. NAM Perspect.

[6] (2018). QuickFacts: Los Angeles County, California. United States Census Bureau.

[7] (2021). Census profile: South Los Angeles, CA. Census Reporter.

[8] (2021). Census profile: East Los Angeles, CA. Census Reporter.

[9] (2019). LGBT Demographic Data Interactive. The Williams Institute.

[10] Wirtz AL, Poteat TC, Malik M, Glass N (2020). Gender-based violence against transgender people in the United States: a call for research and programming. Trauma Violence Abuse.

[11] Balcazar H, Rosenthal E Lee, Brownstein J Nell, Rush CH, Matos S, Hernandez L (2011). Community health workers can be a public health force for change in the United States: three actions for a new paradigm. Am J Public Health.

[12] (2022). Vision y Compromiso.

[13] VaccinateLA (2022). VaccinateLA.

[14] Chan A, Brown Brandon, Sepulveda Enedina, Teran-Clayton Lorena (2015). Evaluation of fotonovela to increase human papillomavirus vaccine knowledge, attitudes, and intentions in a low-income Hispanic community. BMC Res Notes.

[15] Liu M, Chung JE, Li J, Robinson B, Gonzalez F (2022). A case study of community-academic partnership in improving the quality of life for asthmatic urban minority children in low-income households. Int J Environ Res Public Health.

[16] Kim MM, Cheney A, Black A, Thorpe RJ, Cene CW, Dave GJ, Schaal J, Vassar S, Ruktanonchai C, Frerichs L, Young T, Jones J, Burke J, Varma D, Striley C, Cottler L, Brown A, Sullivan G, Corbie-Smith G (2020). Trust in community-engaged research partnerships: a methodological overview of designing a multisite Clinical and Translational Science Awards (CTSA) initiative. Eval Health Prof.

[17] Bright CF, Haynes EE, Patterson D, Pisu M (2017). The value of social network analysis for evaluating academic-community partnerships and collaborations for social determinants of health research. Ethn Dis.

